# Comparative efficacy of scalpel and diode laser techniques in gingival depigmentation: A split-mouth randomized controlled trial with RGB photographic Quantification

**DOI:** 10.1016/j.jobcr.2025.05.001

**Published:** 2025-05-14

**Authors:** Devadharshini Chandrasekar, Pavithra Gopalakrishnan, Vijayalakshmi Rajaram, Burnice Nalina Kumari Chellathurai, Anitha Logaranjani, Jaideep Mahendra, Nikita Ravi

**Affiliations:** Department of Periodontics, Meenakshi Ammal Dental College and Hospital, Chennai, Tamil Nadu, India

## Abstract

**Background:**

Gingival aesthetics, integral to a smile's attractiveness, can be significantly impacted by pigmentation. Melanin, produced by melanocytes, contributes to this pigmentation, which can be addressed through various depigmentation techniques. This study aims to compare the efficacy of conventional scalpel and diode laser methods for gingival depigmentation using RGB photographic analysis.

**Materials and methods:**

This split-mouth, randomized controlled trial involved five participants with bilateral maxillary gingival hyperpigmentation. One sextant per participant was treated with a scalpel (Group 1), and the contralateral sextant was treated with a diode laser (940 nm) (Group 2). Parameters assessed included intraoperative bleeding, postoperative pain (VAS), wound healing (Wound Healing Index), and pigmentation (Dummett Oral Pigmentation Index). RGB photographic analysis was used to quantify colour changes.

**Results:**

No significant difference was observed in intraoperative bleeding between the groups (p = 0.31). Postoperative pain was significantly lower in Group 2 on Day 1 (p = 0.01), though this difference was not significant by Day 7 (p = 0.25). Wound healing scores were comparable at 7 days and 6 months but were significantly better in Group 2 at 12 months (p = 0.01). Pigmentation reduction was significantly greater in Group 2 at 6 months (p = 0.01), but the difference was not significant at 12 months (p = 1.00). RGB analysis revealed that Group 2 achieved superior control of pigmentation, with significant differences in red, green, and blue values at multiple time points (p < 0.001).

**Conclusion:**

Diode laser treatment (Group 2) demonstrated superior aesthetic outcomes and reduced postoperative pain compared to the scalpel technique (Group 1), along with more effective long-term pigmentation control. RGB analysis provided valuable objective data supporting these findings.

## 1 Introduction

The aesthetics of a smile are fundamental to how individuals express joy and approachability, playing a crucial role in social interactions and self-esteem.^1^ While various factors contribute to the overall beauty of a smile, the appearance of the gingiva is often underappreciated yet vital for achieving an appealing smile design. Key aspects such as the contour of the gingival zenith and the presence of pigmentation can significantly influence this aesthetic, particularly for those with pronounced lip lines or a gummy smile.^2^

Melanin, produced by melanocytes, is the primary pigment responsible for gingival coloration. These melanocytes are activated by several stimuli, including environmental factors, leading to the synthesis of melanin through intricate biochemical pathways.^3^ The occurrence of gingival hyperpigmentation varies widely among different demographic groups, often influenced by genetic predisposition and behaviours such as tobacco usage.^4^ To counteract pigmentation, various gingival depigmentation techniques are employed, including traditional scalpel methods, gingivectomy, free gingival grafts, and laser therapy, each aiming to enhance the visual appeal of the gums.^5^

Lasers have become increasingly popular in dental practices since the 1980s, with diode lasers recognized for their efficacy in procedures like frenectomy and gingivectomy. Their advantages include reduced post-operative discomfort and increased precision in surgical interventions.^6^ Lasers offer several advantages in gingival depigmentation, making them a preferred choice in periodontal therapy. Their precision allows for targeted treatment, minimizing damage to surrounding healthy tissues while effectively removing pigmented areas. Additionally, laser procedures typically result in reduced bleeding due to the coagulation of blood vessels during treatment.^7^

RGB photographic analysis serves as a valuable, non-invasive tool for measuring changes in gingival colour post-depigmentation. This technique breaks down images into their red, green, and blue components, allowing for an in-depth evaluation of subtle colour variations and providing a standardized method for comparing treatment outcomes.^8^

The objective of this study is to assess the effectiveness of gingival depigmentation techniques using both traditional scalpel methods and diode laser applications. By integrating RGB photographic analysis, this research aims to deliver a comprehensive, objective comparison of aesthetic results associated with these two treatment modalities.

## 2 Materials and methods

### 2.1 Participant selection

**Fig. 1:**
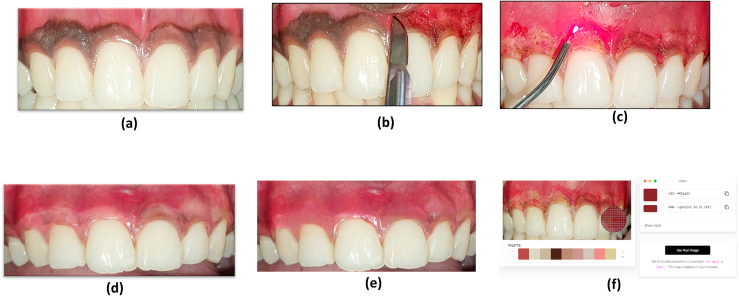
Comparative Clinical and Photographic Evaluation of Gingival Depigmentation Techniques Using Scalpel and Diode Laser Approaches 1a. Pre-operative clinical presentation showing bilateral maxillary gingival hyperpigmentation, highlighting the indication for depigmentation therapy. 1b. Intraoperative view of Group A treated with the conventional scalpel depigmentation technique. 1c. Intraoperative view of Group B managed using a diode laser-assisted depigmentation approach. 1d. Post-operative clinical evaluation on Day 7, demonstrating initial healing outcomes. 1e. Follow-up evaluation at 6 months post-treatment, depicting sustained clinical results. 1f. Photographic analysis with quantitative RGB hue values for objective assessment of pigmentation reduction.

This randomized, split-mouth controlled clinical trial included five participants, selected from patients seeking treatment for gingival depigmentation at the Department of Periodontics, from Meenakshi Ammal Dental College and Hospital. Each participant presented with bilateral maxillary gingival hyperpigmentation suitable for depigmentation treatment (Fig. 1). The study was conducted following the approval of the Institutional Ethical Committee, and all participants provided written informed consent.

### 2.2 Inclusion and exclusion criteria

Inclusion criteria required participants to be systemically healthy individuals aged 18–45 years, with bilateral maxillary gingival hyperpigmentation and no history of previous depigmentation treatments. Only patients with Fitzpatrick skin types I to IV were included to ensure consistent assessment of pigmentation changes. Exclusion criteria eliminated participants with smoking habits, pregnancy or lactation, systemic diseases such as diabetes, immune disorders, or any condition affecting wound healing, and those using medications that could interfere with healing or pigmentation outcomes.

### 2.3 Randomization and study design

This study employed a split-mouth design, with the maxillary gingiva divided into two sextants for each participant. Random allocation, determined by a computer-generated randomization schedule, assigned one sextant to Group 1 (scalpel depigmentation) and the other to Group 2 (diode laser treatment at 940 nm). The left or right sextant was treated with the scalpel technique in half of the cases, with the alternate sextant receiving diode laser treatment. The same operator performed all procedures to minimize variability.

### 2.4 Treatment protocol

For the scalpel group, depigmentation was carried out using a Bard-Parker blade no. 15 under local anaesthesia, with care taken to remove the pigmented epithelial layer and a portion of the underlying connective tissue. The laser group underwent depigmentation using a diode laser (940 nm), set at 1.5 W in continuous mode, with a defocused beam to ablate the pigmented tissue. Post-operatively, participants were instructed on maintaining oral hygiene, and no additional medications or topical agents were applied to influence healing. The patient was recalled for review at Day 7, 3 months and at 6 months (Fig. 1).

### 2.5 Parameters evaluated

The following clinical parameters were assessed to compare the efficacy of both techniques:

1. **Bleeding During Surgery:** Recorded intra-operatively by visual estimation of bleeding severity using a standardized bleeding score.
2. **Wound Healing Index:** Post-operative healing was evaluated at 7 days, 3 months, and 6 months using the Wound Healing Index (WHI), which scores epithelialization, absence of infection, and tissue integrity.
3. **Visual Analog Scale (VAS):** Participants rated their pain perception during and after surgery using a VAS, with scores ranging from 0 (no pain) to 10 (extreme pain).
4. **Gingival Pigmentation and Repigmentation:** The Dummett Oral Pigmentation Index (DOPI) was employed to assess gingival pigmentation pre-operatively, and at 7 days, 3 months, and 6 months post-treatment. Repigmentation was monitored and recorded using the DOPI scale to determine the recurrence of pigmentation.

### 2.6 RGB photographic analysis

High-resolution digital photographs of the treated gingiva were taken at baseline, 7 days, 3 months, and 6 months post-treatment. These images were analyzed using Adobe Photoshop software, which utilizes a red, green, and blue (RGB) value system to quantify the color changes in the gingiva post-depigmentation.

• **Red Channel:** Assessed haemoglobin concentration in the gingiva, providing insight into vascular healing and inflammation levels.
• **Green Channel:** Represented the cytoplasmic tissue, indicating cellular composition and tissue health.
• **Blue Channel:** Measured the presence of dark pigmentation, corresponding to melanin levels in the gingiva.

RGB analysis was used to objectively evaluate the effectiveness of each technique in reducing pigmentation and promoting a uniform appearance. This method allowed for detailed color channel breakdowns, providing quantitative data to compare the aesthetic outcomes of the scalpel and laser treatments. Colour values were recorded and compared between baseline, 7 days, 3 months, and 6 months to assess long-term efficacy.

## 3 Statistical analysis

The data collected from clinical evaluations were entered into a Microsoft Excel spreadsheet and subjected to statistical analysis using the Statistical Package for Social Sciences (SPSS) software, version 24 (IBM, Chicago, USA). Continuous variables, such as the RGB values, VAS scores, and Wound Healing Index, were expressed as either mean ± standard deviation (SD) or median ± interquartile range (IQR), depending on data distribution.

To determine the normality of the data, the Shapiro-Wilk test was applied. Given that most variables did not follow a normal distribution, non-parametric tests were utilized for further statistical comparisons. The Mann-Whitney *U* test was employed for intergroup comparisons between the scalpel and diode laser groups at each evaluation point (baseline, 7 days, 3 months, and 6 months). For intra-group comparisons of the same parameter across different time points, the Friedman test was used.

All tests were two-sided, and a p-value of less than 0.05 was considered statistically significant. This approach allowed for rigorous comparison of the clinical outcomes and ensured the robustness of the statistical findings throughout the study period.

## 4 Results

### 4.1 Bleeding During Surgery

The comparison of intra-operative bleeding between the groups demonstrated no statistically significant difference (p = 0.31) (Table 1). The observed mean bleeding scores were slightly lower in the Group 2, although the difference did not reach statistical significance.0.

**Table 1:** Intergroup comparison of bleeding during surgery between.

Bleeding during surgery	Group	N	Mean ± Std. Deviation	p-value^a^
	**Scalpel** (Group 1)	5	1.4 ± 0.54	0.31
	**Laser** (Group 2)	5	1.0 ± 0.0	

a Mann- Whitney *U* test.

### 4.2 Visual Analog Scale (VAS)

Assessment of post-operative pain using the Visual Analog Scale (VAS) revealed a significant difference between the groups on Day 1, with the group 2 reporting lower pain levels compared to the group 1 (p = 0.01). By Day 7, however, the difference in VAS scores was not statistically significant (p = 0.25) (Table 2).

**Table 2:** Intergroup comparison of Visual Analog Score (VAS).

	Group	N	Mean ± Standard Deviation	p-value^a^
VAS after Day 1	Group 1 (Scalpel)	5	15.0 ± 5	**0.01**^b^
	Group2 (Laser)	5	10.0 ± 0	
VAS after Day 7	Group 1 (Scalpel)	5	8.0 ± 2.73	0.25
	Group2 (Laser)	5	6.0 ± 2.23	

a Mann- Whitney *U* test.

b p-value<0.05- statistically significant.

### 4.3 Wound healing

Wound healing was evaluated at 7 days, 6 months, and 12 months post-operatively. At 7 days and 6 months, there were no significant differences in wound healing scores between the groups 1 and 2 (p = 0.31 and p = 0.69, respectively). At 12 months, group 2 exhibited significantly better wound healing compared to group 1 (p = 0.01(Table 3).

**Table 3:** Intergroup comparison of Wound healing between Scalpel and Laser.

	Group	N	Mean ± Standard Deviation	p-value^a^
Wound Healing after 7 days	Group 1 (Scalpel)	5	2.2 ± 0.44	0.31
	Group2 (Laser)	5	2.6 ± 0.54	
Wound healing after 6 months	Group 1 (Scalpel)	5	2.8 ± 0.44	0.69
	Group2 (Laser)	5	3.0 ± 0.70	
Wound healing after 12 months	Group 1 (Scalpel)	5	4.0 ± 0	**0.01**^b^
	Group2 (Laser)	5	4.6 ± 0.58	

a Mann- Whitney *U* test.

b p-value<0.05- statistically significant.

### 4.4 Gingival pigmentation

Gingival pigmentation was assessed using the Dummett Oral Pigmentation Index (DOPI). Initial assessments at 7 days post-operatively showed no significant differences between both the groups (p = 0.31). However, by 6 months, group 2 exhibited significantly lower pigmentation scores compared to the group 1 (p = 0.01), indicating more effective pigmentation reduction. At 12 months, pigmentation levels were comparable between both the groups (p = 1.00) (Table 4).

**Table 4:** Intergroup comparison of pigmentation between Scalpel and Laser.

	Group	N	Mean ± Standard Deviation	p-value^a^
Pigmentation after 7 days	Group 1 (Scalpel)	5	1.8 ± 0.44	0.31
	Group2 (Laser)	5	1.4 ± 0.54	
Pigmentation after 6 months	Group 1 (Scalpel)	5	2.0 ± 0.0	**0.01**^b^
	Group2 (Laser)	5	1.0 ± 0.0	
Pigmentation after 12 months	Group 1 (Scalpel)	5	1.4 ± 0.54	1.00
	Group2 (Laser)	5	1.4 ± 0.54	

a Mann- Whitney *U* test.

b p-value<0.05- statistically significant.

### 4.5 RGB photographic analysis

RGB photographic analysis was employed to evaluate the changes in pigmentation using Adobe Photoshop. Table 5 presents the intergroup comparison of Red, Green, and Blue values at pre-operative and immediate post-operative intervals. The group 2 showed a statistically significant increase in Red values immediately post-surgery (p < 0.001), suggesting better control of bleeding. Significant differences in Green and Blue values were also observed, with the group 2 demonstrating improved outcomes (p < 0.001).

**Table 5:** Intergroup comparison of red, green, blue values between scalpel and laser group at baseline, 7 days, and 6 Months.

Time Point	Parameter	Group	N	Mean ± S.D	Median ± IQR	p-value
**Baseline**	**Red (Pre-OP)**	**Scalpel (Group 1)**	**5**	**90 ± 4.18**	**88 ± 8.0**	**0.85**
		**Laser (Group 2)**	**5**	**90.6 ± 5.94**	**92 ± 11.5**	
	**Green (Pre-OP)**	**Scalpel (Group 1)**	**5**	**56.2 ± 2.77**	**57 ± 5.0**	**0.02∗**
		**Laser (Group 2)**	**5**	**62.4 ± 4.03**	**63 ± 7.5**	
	**Blue (Pre-OP)**	**Scalpel (Group 1)**	**5**	**52.4 ± 2.88**	**53 ± 5.5**	**0.65**
		**Laser (Group 2)**	**5**	**55.6 ± 15.15**	**61 ± 20.5**	
**7 Days**	**Red**	**Scalpel (Group 1)**	**5**	**144.6 ± 3.05**	**145 ± 6.0**	**<0.001∗∗**
		**Laser (Group 2)**	**5**	**155.4 ± 2.07**	**156 ± 3.5**	
	**Green**	**Scalpel (Group 1)**	**5**	**83.8 ± 3.03**	**84 ± 5.5**	**0.53**
		**Laser (Group 2)**	**5**	**85.0 ± 2.82**	**86 ± 5.5**	
	**Blue**	**Scalpel (Group 1)**	**5**	**88.0 ± 2.23**	**88 ± 4.0**	**<0.001∗∗**
		**Laser (Group 2)**	**5**	**92.2 ± 2.77**	**93 ± 5.0**	
**6 Months**	**Red**	**Scalpel (Group 1)**	**5**	**125.2 ± 2.77**	**126 ± 5.0**	**<0.001∗∗**
		**Laser (Group 2)**	**5**	**174.2 ± 2.88**	**174 ± 5.5**	
	**Green**	**Scalpel (Group 1)**	**5**	**62 ± 2.55**	**62 ± 5.0**	**<0.001∗∗**
		**Laser (Group 2)**	**5**	**103.8 ± 2.28**	**104 ± 4.5**	
	**Blue**	**Scalpel (Group 1)**	**5**	**64.2 ± 2.28**	**64 ± 4.5**	**<0.001∗∗**
		**Laser (Group 2)**	**5**	**101.8 ± 1.48**	**102 ± 2.5**	

Note.

*Man–*Whitney *U* test.

p-value <0.05 – statistically significant.

p-value <0.001 – highly statistically significant.

At 7 days post-operatively, group 2 maintained significantly higher Red and Blue values compared to the group 1 (p < 0.001), reflecting superior pigmentation control and healing. These differences remained statistically significant at 6 months for all color channels (p < 0.001), indicating sustained benefits of the laser technique.

**Fig. 2:**
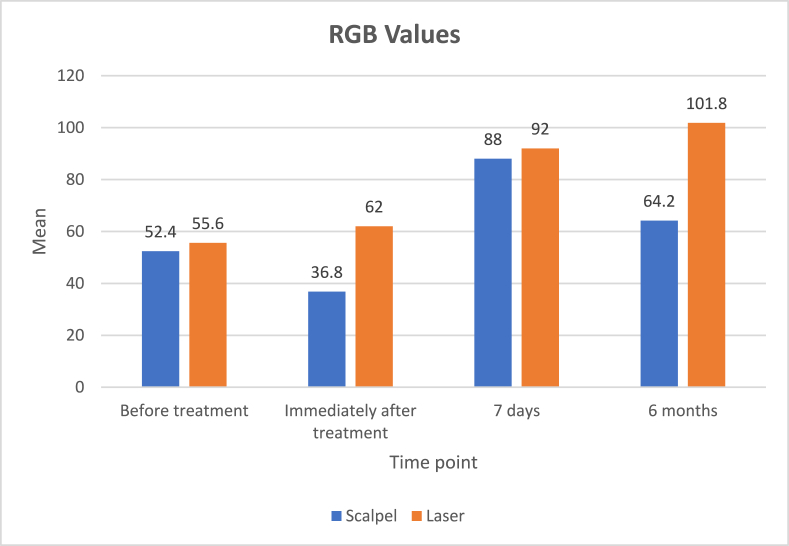
Comparison of RGB values between scalpel and laser groups at various time points.

Intragroup comparisons within the scalpel and laser groups reveal that the laser group experienced continuous improvements in RGB values over time (p = 0.03), suggesting more effective and long-lasting pigmentation reduction compared to the scalpel technique (Fig. 2).

## 5 Discussion

The comparison of gingival depigmentation techniques utilizing scalpel and diode laser methods reveals key insights into both clinical efficacy and patient comfort. The choice of method for gingival depigmentation is often influenced by the desired aesthetic outcome, patient tolerance, and long-term stability of results.

The study demonstrated a significant reduction in postoperative pain for the laser group on the first day following treatment, as evidenced by the lower VAS scores (p = 0.01). This outcome aligns with findings from previous research, El Shenawy et al. which suggests that diode lasers are associated with reduced postoperative discomfort due to their ability to coagulate blood vessels and minimize inflammation. In contrast, the scalpel method, while effective, may cause greater tissue trauma, leading to increased pain during the initial postoperative period.^9^ However, by Day 7, the pain levels between the two groups became comparable (p = 0.25), suggesting that the early advantage provided by the laser is primarily due to its less invasive nature during the initial healing phase.

The assessment of wound healing using the Wound Healing Index revealed that both techniques facilitated satisfactory healing at 7 days and 6 months postoperatively, with no significant differences (p = 0.31 and p = 0.69, respectively). This finding is consistent with the findings of Abdelmagyd et al. the scalpel technique demonstrates notable superiority in the treatment of gingival hyperpigmentation compared to laser and abrasion methods, due to its effectiveness, minimal time requirement, ease of execution, and lower recurrence rates, making it a viable option for long-term clinical success,^10^ indicating that scalpel techniques can achieve effective healing, provided proper postoperative care is maintained. However, at 12 months, the laser group showed superior wound healing (p = 0.01), a result likely attributed to the laser's ability to promote tissue regeneration and minimize bacterial contamination during healing. This aligns with the study by Mikhail et al. highlights that while diode laser is an effective and efficient tool for gingival depigmentation, yielding minimal pain and reduced bleeding.^11^ This long-term benefit underscores the potential of laser therapy to deliver sustained aesthetic and clinical outcomes, particularly in procedures where minimizing repigmentation is a primary concern.

A key factor in evaluating the success of gingival depigmentation is the ability to control pigmentation recurrence. The laser technique achieved significantly lower pigmentation scores at the 6-month evaluation (p = 0.01), a finding that mirrors previous research advocating lasers as a superior modality for managing gingival hyperpigmentation due to their selective targeting of melanocytes. The Dummett Oral Pigmentation Index (DOPI) findings further confirmed that, although pigmentation levels between the groups became comparable by 12 months (p = 1.00), the laser group exhibited more efficient pigmentation reduction in the intermediate term. This is consistent with the findings of Prasanth et al., where the diode laser technique demonstrated superior outcomes in managing gingival hyperpigmentation compared to the scalpel technique, particularly in terms of operator convenience, reduced postoperative bleeding, and delayed repigmentation, despite the noted thermal effects on adjacent tissues that resulted in slower healing.^12^ This finding contradicts the results of the study by Bakutra et al. which reported that surgical stripping demonstrated significantly less repigmentation and superior outcomes compared to diode laser ablation for gingival depigmentation, as evidenced by both clinical parameters and immunohistological analysis.^13^

The use of RGB photographic analysis introduced a novel, objective, and quantifiable method for evaluating aesthetic outcomes in gingival depigmentation. Since the mid-1990s, digital photography has been increasingly integrated into clinical dental practice, aided by advancements in digital camera technologies and analytical software. Software like Adobe Photoshop facilitates image standardization, measurement, and enhancement, with its RGB (Red, Green, Blue) color model offering precise hue analysis. In this model, blue hues represent melanin-associated darker pigmentation, red corresponds to hemoglobin levels, and green reflects cytoplasmic content.^14^

RGB analysis allows clinicians to evaluate subtle pigmentation changes over time with greater precision than visual inspection alone. This study demonstrated that the diode laser group consistently showed higher red and green values, and lower blue values compared to the scalpel group—indicative of lighter gingival tone and reduced melanin presence. These changes translated into visible aesthetic improvements, reinforcing the clinical utility of digital photographic evaluation in both monitoring and documenting depigmentation therapy outcomes.

Our findings are consistent with those of Shah et al. who also reported that while both scalpel and laser treatments were effective, the diode laser provided added advantages in terms of reduced postoperative discomfort and faster recovery. By incorporating RGB hue analysis, our study not only supports their conclusions but also highlights the benefit of digital assessment tools as adjuncts to traditional clinical evaluations. This approach enhances clinician-patient communication and supports evidence-based aesthetic dentistry.^8^

Importantly, by the 12-month follow-up, pigmentation levels in both groups converged, suggesting that long-term relapse may occur independent of the technique used. This finding implies that individual melanin activity, habits such as smoking, or systemic influences may contribute to repigmentation. Hence, regular monitoring and possible maintenance therapy may be necessary for sustained aesthetic results.

This study stands out for its innovative integration of digital RGB photographic analysis as a quantitative tool in evaluating the outcomes of gingival depigmentation procedures. While conventional depigmentation studies primarily rely on clinical observation and subjective color grading, our methodology introduces a more objective, reproducible, and standardized approach to documenting aesthetic outcomes. By employing Adobe Photoshop's RGB value model, we precisely measured hue variations associated with melanin reduction, thereby bridging the gap between clinical dentistry and digital technology. To the best of our knowledge, this is among the few studies to directly correlate RGB-derived colorimetric data with clinical healing trajectories across two distinct surgical modalities scalpel and diode laser. This digital integration provides a scalable framework for future aesthetic dentistry research, enabling clinicians to track pigmentation changes with greater accuracy and consistency.

### 5.1 Strengths of the study

A significant strength of this study lies in its use of objective digital image analysis to quantify pigmentation changes. The RGB system allowed for a scientifically robust assessment that reduces subjective bias and enhances repeatability. Furthermore, the split-mouth design minimized interindividual variability and allowed for a more reliable intra-patient comparison between the two techniques. The inclusion of a 12-month follow-up period is another notable advantage, enabling the evaluation of long-term stability and recurrence of pigmentation, which is critical for assessing treatment durability. Importantly, the study also offers clinically relevant insights by correlating RGB differences with visible changes in gingival aesthetics, helping practitioners translate quantitative findings into practical decisions during treatment planning.

### 5.2 Limitations of the study

Despite its strengths, the study has certain limitations that warrant consideration. The relatively small sample size may affect the generalizability of the findings, particularly in diverse populations with varying melanin expression patterns. Moreover, the accuracy of RGB analysis is highly contingent on the standardization of photographic parameters such as lighting, angle, and camera settings, introducing potential variability that could influence results. The focus on the maxillary anterior region alone limits extrapolation of outcomes to other intraoral sites, where tissue characteristics and healing responses may differ. Additionally, the study did not incorporate patient-reported outcomes related to aesthetic satisfaction. Including such subjective evaluations in future studies could provide a more comprehensive understanding of treatment success from both clinical and patient perspectives.

## 6 Conclusion

In conclusion, diode laser treatment outperformed traditional scalpel techniques in both aesthetic and clinical outcomes. It resulted in reduced postoperative pain and improved long-term pigmentation control, as supported by objective RGB analysis. The long-term results, supported by objective RGB analysis, demonstrate that diode laser therapy provides a more uniform and stable gingival color, crucial for aesthetic outcomes in cosmetic procedures. The RGB analysis highlights the precision and reliability of the laser in maintaining consistent pigmentation, emphasizing its potential in optimizing gingival aesthetics.These findings highlight the diode laser's potential as a valuable advancement in gingival aesthetics, offering precise, reliable, and cosmetically superior results in periodontal procedures. This technology presents a promising alternative for achieving optimal clinical and aesthetic outcomes in gingival treatments.

## Statement of informed consent

This is to certify that all patients participating in the study titled **“Comparative Efficacy of Scalpel and Diode Laser Techniques in Gingival Depigmentation: A Split-Mouth Randomized Controlled Trial with RGB Photographic Quantification”** were provided with a detailed consent form.

The objectives, procedures, potential risks, and benefits of the study were thoroughly explained to each participant in a language they understood. Adequate time was given to address any questions or concerns raised by the patients.

All participants voluntarily agreed to participate in the study and signed the consent form prior to their inclusion. A copy of the signed consent form was provided to each patient for their records.

## Ethical clearance statement

This study, titled **“Comparative Efficacy of Scalpel and Diode Laser Techniques in Gingival Depigmentation: A Split-Mouth Randomized Controlled Trial with RGB Photographic Quantification,”** was conducted in full compliance with ethical principles outlined in the Declaration of Helsinki.

Prior to the initiation of the study, ethical clearance was obtained from the Institutional Ethics Committee of Meenakshi Ammal Dental College and Hospital, Chennai, Tamil Nadu, India. The committee reviewed and approved the study protocol under the reference number **[MADC/IEC/II/31-B/2024].**

All participants were informed about the purpose, procedures, potential risks, and benefits of the study, and written informed consent was obtained from each participant before enrollment. Confidentiality and anonymity of participant data have been strictly maintained throughout the study.

We confirm that the study adheres to the ethical standards required for human research and complies with all relevant institutional and national guidelines.

## Statement

This research did not receive any specific grant from funding agencies in the public, commercial, or not-for-profit sectors.

All expenses related to the study, including materials, equipment, and analysis, were funded by the authors themselves.

If additional clarifications regarding funding are required, please feel free to contact:

## Declaration of competing interest

The authors declare that they have no known competing financial interests or personal relationships that could have appeared to influence the work reported in this paper.
